# Test–retest reliability of mandibular morphology measurements on cone-beam computed tomography-synthesized cephalograms with random head positioning errors

**DOI:** 10.1186/s12938-017-0353-8

**Published:** 2017-05-30

**Authors:** Hsien-Shu Lin, Yunn-Jy Chen, Hsuan-Lun Lu, Tung-Wu Lu, Chien-Chih Chen

**Affiliations:** 1 Department of Dentistry, School of Dentistry, National Taiwan University Hospital, Taipei, Taiwan, ROC; 2 Institute of Biomedical Engineering, National Taiwan University, Taipei, Taiwan, ROC; 3 Department of Orthopaedic Surgery, School of Medicine, National Taiwan University, Taipei, Taiwan, ROC; 4 Department of Dentistry, Cardinal Tien Hospital, New Taipei City, Taiwan, ROC

**Keywords:** Intra-class correlation coefficient (ICC), Mandible, Reliability, Simulated radiograph, Head positioning

## Abstract

**Background:**

Cephalometric radiography has been used for orthodontic and surgical treatment planning and assessment, and for quantifying mandibular growth. However, it remains unclear how head positioning errors and the level of examiner experience affect the reliability of such morphometric measurements. The current study aimed to bridge the gap by determining the intra-, inter-rater, and inter-session reliability of measurements of mandibular morphology with random head positioning errors as measured by a junior and a senior dentist.

**Methods:**

Cone-beam computed tomography data of twelve mandibles were obtained with each rotated randomly away from the neutral position within the range of +3 and −3° along each of the anatomical axes to simulate six imaging trials. A synthetic cephalogram for each trial was obtained via a digitally reconstructed radiography (DRR) technique and eleven landmarks for twelve morphological parameters on the cephalogram were identified manually six times by a junior and a senior dentist. The procedure was repeated on another day within 5 days. Test–retest reliability was assessed in terms of an intra-class correlation coefficient (ICC) using a two-way mixed-effects model.

**Results:**

Good to very good intra-rater (senior: ICC > 0.92; junior: ICC > 0.78), inter-rater (ICC > 0.70 for most parameters) and inter-session reliability (senior: ICC > 0.84; junior: ICC > 0.62) were found. Bland & Altman plots of inter-rater comparisons show that there were systematical biases between the examiners on most parameters, except for the distance between Gonion and Pogonion.

**Conclusions:**

The current results suggest that good to very good intra-rater, inter-rater and inter-session reliability can be achieved for most parameters with randomized head positioning errors; higher inter-session reliability can be achieved by more experienced examiners; and that long-term monitoring of mandibular growth based on cephalographic measurements should be made by the same more experienced examiner. The current DRR-based approach can be used to evaluate individual factors that affect the morphological measurements.

## Background

Cephalometric radiography and analysis have been used for orthodontic and surgical treatment planning and assessment, and for studying the dental-skeletal relationship since first introduced by Broadbent in 1981 [1, 2]. Cephalometric radiographs have also been used to measure morphological parameters that quantify the growth of the mandible, giving useful information for orthodontic treatment or craniofacial surgery [3, 4]. Although easy to take and analyze clinically, cephalometric radiographs are subject to errors owing to malpositioning of the bone in the three-dimensional (3D) space, and thus the X-ray projection onto the image plane to form the two-dimensional (2D) radiograph. Since the X-ray beams diverge, the bone under examination at different positions relative to the image plane will produce images of different sizes, positions and intensities, leading to errors in the measurements, and thus their subsequent interpretation [5, 6].

Positioning of the head, and thus the mandible during imaging, is an important factor among others that affect radiogram-based measurements of the mandible, such as identification of bony landmarks, examiner experience, and superimposition of the craniofacial structures, leading to biased examination outcomes [7]. Therefore, the reliability of cephalometric measurements relies heavily on the ability to use a standardized, reproducible head positioning procedure in relation to the X-ray source and image plane [8]. Previous studies have shown that accurate positioning of the head in a neutral position can lead to highly reliable mandibular measurements with high intra-class correlation coefficient (ICC) values [9]. However, accurate positioning of the head is not as straightforward as it appears. While positioning devices such as ear rods and nasal positioners could be used for head positioning, it is difficult to achieve perfect head alignment for various reasons, including anatomical variations of the head. Several studies have shown that head rotation away from the neutral position can affect landmark identification, and thus the accuracy of cephalometric measurements [10–16]. Malpositioning of the head may also affect the judgment of clinicians with different levels of experience when carrying out the morphological measurements at different stages in the management of a patient. Therefore, it is necessary to determine whether the morphological measurements made on the cephalograms are reliable both within (intra-rater) and between clinicians (inter-rater), and between sessions (inter-session) subject to the uncertainties of head positioning.

Studies on the reliability of mandibular morphological measurements on planar radiographs have been limited. Most studies have focused on the intra- and inter-rater reliability of identifying the anatomical landmarks that define the mandibular morphology on 2D radiographs in terms of ICC values [17, 18]. Few studies have evaluated quantitatively the intra-rater, inter-rater and inter-session reliability of the determined morphological parameters of the human mandible from 2D radiographs. Furthermore, no study has quantified the effects of the identification errors in bony landmarks on the reliability of morphological parameter determination under random errors in head positioning during imaging. It is also unclear how the levels of experience of the examiners would affect the reliability of morphological measurements with random head positioning errors.

In recent years, cone-beam computed tomography (CBCT) has been used widely for orthodontic analysis [19, 20] and craniofacial surgery [21, 22]. By taking advantage of computer simulations, the CBCT data can be used to generate 2D synthesized cephalograms using the technique of digitally reconstructed radiographs (DRR) on which repeated planar measurements are carried out to determine the reliability of the measurements. The DRR technique has been used to study the morphology of human ankles [23], canine hips [24] and mandibles [9, 25]. With this technique the effects of errors in identifying bony landmarks on the reliability of determining morphological parameter with random head positioning errors could be studied by simulating random head positions without the effects of other factors such as superimposition of craniofacial structures.

The purposes of the current study were to determine the intra-, inter-rater, and inter-session reliability of determining morphological parameters of the human mandible on CBCT-synthesized cephalometric radiographs with random head positioning errors, and to identify the effects of examiner experience on the above reliability measures.

## Methods

Twelve subjects (age: 37 ± 7 years; 6 males, 6 females) scheduled for orthodontic evaluation participated in the current study with informed written consent as approved by the Ethics Committee of Cardinal Tien Hospital, Taipei, Taiwan (IRB Number: CTH-100-2-5-038). Each subject received a CBCT scan of the mandibular area by a CBCT system (i-CAT, Xoran Technologies, Ann Arbor, USA) with a slice thickness of 0.4 mm and an intra-slice pixel size of 0.4 mm × 0.4 mm. The CBCT scanning took 20 s, and, according to the manufacturer, had a radiation dose of 68 Sv at a tube current of 18.45 mAs and a tube potential of 120 kVp. This is comparable to the dose previously measured for the maxillofacial region [26].

**Fig. 1:**
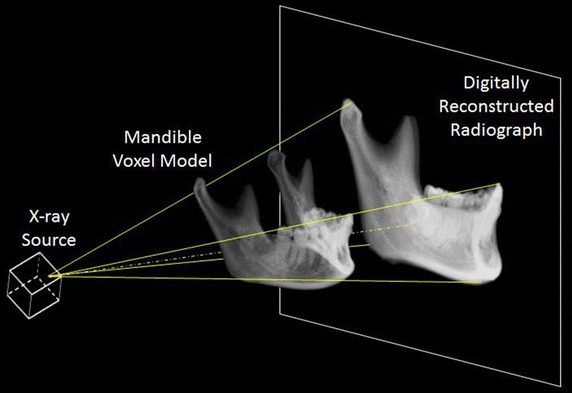
Generation of a synthetic cephalogram image of the mandible from the CBCT volume of the mandible using a digitally reconstructed radiography (DRR) technique. The radiography system was modeled as an ideal perspective projection of a point source X-ray through the bone onto the image plane. The CBCT volume of the mandible was positioned within the radiographic projection model with the principal axis of the projection defined as the line connecting the most prominent points on the medial surfaces of the bilateral condyles

For each subject, the mandible within the CBCT volume was segmented using a region-growing with thresholds method (Amira, Visage Imaging Inc., Germany) to determine the surface of the mandible (Fig. 1). The epicondyles and the centers of the edges of the two central incisors on the surface were digitized manually by an experienced dentist (LHS) using Geomagic 3D (Geomagic, Inc., USA). The reliability of this procedure was determined by repeated identifications of the landmarks by the same dentist, giving an intra-class correlation coefficient (ICC) of 0.9, which was considered strong for the current purpose. These were then used to define an anatomical coordinate system (ACS) embedded within the CBCT volume of the mandible. The ACS originated at the mid-point between the epicondyles, with the z-axis directed to the right epicondyle, the y-axis directed superiorly and normal to the plane defined by the z-axis and the mid-point of the centers of the central incisor edges, and the x-axis as the cross-product of y-axis and z-axis, and directed anteriorly [27].

The synthetic cephalogram image of the mandible was generated from the CBCT volume of the mandible using a digitally reconstructed radiography (DRR) generation technique [28], which modeled the radiography system as an ideal perspective projection of a point source X-ray through the bone onto the image plane. In the current study, the radiographic projection model was set up to model a commercially available cephalogram system used in the authors’ hospital, namely an Orthoceph OC 100 X-ray system (Instrumentarium Corporation, Imaging Division, Tuusula, Finland). The CBCT volume of the mandible was positioned within the radiographic projection model with the principal axis of the projection defined as the line connecting the most prominent points on the medial surfaces of the bilateral condyles. The X-ray source was positioned on the right side of the mid-sagittal plane of the mandible at a distance of 1520 mm, while the image plane was located 152 mm away from the left side of the mid-sagittal plane, opposite the source. This position of the CBCT volume of the mandible is here referred to as the neutral position. For testing the effects of head malpositioning on the reliability of morphological measurements, the CBCT volume of the mandible for each subject was rotated randomly away from the neutral position within a range of +3 and −3° about the x-, y-, and z-axis. A total of six random rotational displacements were imposed for each subject, simulating six independent imaging trials. For each perturbed position, the DRR-based cephalograms were generated by casting rays from the model point-source X-ray through the CBCT volume of the mandible using a ray-tracing with tri-linear interpolation method [29], giving 2D images with a pixel size of 0.29 mm × 0.29 mm.

**Fig. 2:**
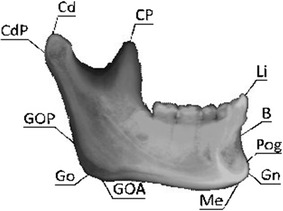
Bony landmarks on the CBCT-synthesized cephalogram of the mandible. Definitions of the landmarks and parameters are referred to in Table 1

Two dentists, one with 11 years and the other with 1 year of experience, were recruited from National Taiwan University Hospital to participate as examiners in the current study. For each of the subjects, his/her synthetic cephalograms were presented to both of the examiners, one at a time in a random order. On each cephalogram the examiners were asked to identify eleven landmarks that describe the key morphological features of the mandible (Table 1; Fig. 2) using a mouse pointer with the assistance of a graphics-based user interface implemented in MATLAB (MathWorks, Inc., USA) on a personal computer. Each anatomical landmark was identified six times (trials) by each examiner (Fig. 2). The re-test was performed at approximately the same time of the day on a subsequent day within a period of 5 days after the first session, following the same test procedure. For each trial the identified landmarks were used to define line segments that were used to calculate a total of 12 morphometric parameters describing the growth of the mandible (Table 2), similar to the parameters considered in a previous study [30].

**Table 1:** Definitions of the anatomical landmarks of the mandible necessary for determining the morphological parameters

Bony landmark		Definition
Cd	*Condyle*	The most protruding point on the top of the mandibular condyle
CdP	*Condyle posterior point*	The most posterior protruding point of the mandibular condyle
GOP	*Gonion posterior point*	The most posterior protruding point of the ramus above the gonion
GO	*Gonion*	The midpoint of the contour connecting the ramus and body of the mandible
GOA	*Gonion anterior point*	The most protruding point of the mandible before the gonion
Me	*Menton*	The center of the inferior border on the mandibular symphysis
Pog	*Pogonion*	The most anterior point on the contour of the chin
Gn	*Gnathion*	The center of the inferior border on the mandibular symphysis
B	*B point*	The innermost point on the contour of the mandible between the incisor and the bony chin
Li	*Lower central incisor edge*	The incisal edge of the mandibular central incisor
CP	*Coronid process*	The top point of the coronid process

Morphological parameters	Definition
Cd-Gn, Cd-B, Cd-Li	Parameters related to the changes of the total mandibular length
GO-Pog, GO-Gn, Me-GOA	Parameters related to the changes of the mandibular corpus length
Cd-GO, CdP-GOP, Cd-CP	Parameters related to the changes of the mandibular ramus length
Li-Me	The anterior length of the mandible
Cd-GO-Gn	Gonion angle
GO-Gn-Li	The angle of the lower anterior teeth

**Table 2:** The mean (standard deviation) of each mandibular morphometric parameter for randomized head positions, as well as the intra-rater and inter-rater reliability in terms of intra-class correlation coefficients (ICC) and coefficient of variation (CV) for the senior and junior examiner

	Senior			Junior			Inter-rater	
	Mean	ICC_1_	CV	Mean	ICC_1_	CV	ICC_2_	*p* value
	(SD)			(SD)				
Cd-Gn	106.97 (4.88)	1.00	0.05	104.43 (5.28)	0.98	0.05	0.93	0.000*
Cd-B	95.05 (3.64)	0.99	0.04	93.64 (3.71)	0.96	0.04	0.94	0.000*
Cd-Li	89.46 (4.16)	0.99	0.05	87.65 (3.82)	0.95	0.04	0.92	0.000*
Go-Pog	69.59 (4.42)	0.98	0.06	69.47 (4.77)	0.94	0.07	0.96	0.571
Cd-Go	57.36 (4.15)	0.98	0.07	57.96 (3.87)	0.91	0.07	0.95	0.119
Go-Gn	68.55 (4.34)	0.98	0.06	66.88 (4.92)	0.94	0.07	0.89	0.000*
CdP-GoP	38.02 (3.33)	0.92	0.09	41.00 (3.44)	0.78	0.08	0.60	0.000*
Me-GoA	58.14 (4.53)	0.97	0.08	54.38 (5.55)	0.90	0.10	0.70	0.000*
Cd-CP	33.38 (2.80)	0.98	0.08	32.91 (2.92)	0.94	0.09	0.96	0.074
Li-Me	37.53 (4.74)	0.99	0.13	36.79 (4.68)	0.98	0.13	0.99	0.000*
∠Cd-GO-Gn	116.41 (6.54)	0.99	0.06	113.68 (6.38)	0.96	0.06	0.93	0.000*
∠GO-Gn-Li	79.51 (8.02)	0.99	0.10	81.31 (8.72)	0.97	0.11	0.96	0.002*

The *p* values with an asterisk (*) calculated from the paired *t* test indicate that the differences in the measurement between examiners is less than 0.05

*ICC*_*1*_ Intra-rater correlation coefficient, *ICC*_*2*_ Inter-rater correlation coefficient

*p* value: p values of the paired-sample *t* test of the measurements between senior and junior

* Significant difference in the measurement between examiners

For each morphometric parameter, coefficients of variation (CV) and intra-class correlation (ICC) calculated using a two-way mixed-effects model (ICC3,1) were used to assess the intra-rater and inter-session test–retest reliability, while the inter-rater test–retest reliability was quantified using CV and ICC from a two-way random-effects model (ICC2, k) [31]. ICC values ranging from 0.81 to 1.00 indicate very good reliability; 0.61–0.80 good reliability; 0.41–0.60 moderate reliability; 0.21–0.40 fair reliability; and below 0.2 poor reliability [32]. A paired *t* test was used to compare the differences in measurements between examiners and sessions for each parameter. Bland & Altman plots [33] were used to visualize differences between the two examiners (sessions) against the corresponding mean of the two examiners (sessions) for each subject, enabling the evaluation of the bias between the mean differences, and the estimation of an agreement interval, within which 95% of the differences of the second examiner (session) fall as compared to the first examiner (session). A one-sample *t* test was used to test whether the differences were significantly different from zero. A significance level of 0.05 (α = 0.05) was set for all tests. The values of each morphometric parameter measured from the 12 DRR-synthesized cephalograms were ensemble-averaged across all subjects for each examiner, giving means and standard deviations (SD). All statistical analyses were performed using SPSS version 19.0 (IBM Corp., Armonk, USA).

## Results

Very good intra-rater reliability by the senior examiner was found for all the parameters with ICC values greater than 0.92. Similar results were also found for the junior examiner except that only good reliability was achieved for CdP-GoP (ICC = 0.78, Table 2). Mean CV values were smaller than 0.13 for all the parameters, for both examiners (Table 2).

**Fig. 3:**
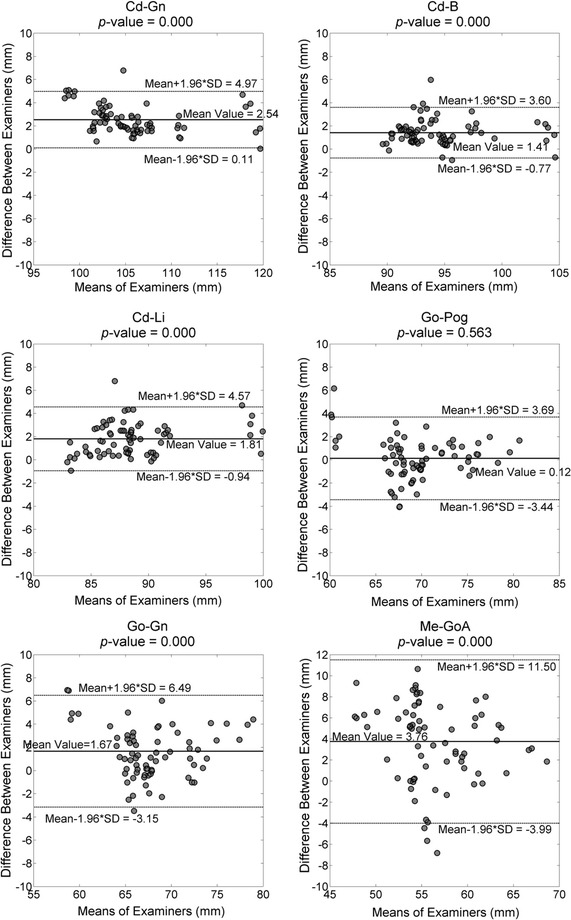
Bland & Altman plots of measurements of each of the mandibular parameters (Cd-Gn, Cd-B, Cd-Li, GO-Pog, GO-Gn, Me-GOA) by the two examiners for randomized head positions. The *solid lines* indicate the mean values of both examiners. The *dashed lines* indicate the 95% confident interval of the difference between the examiners

**Fig. 4:**
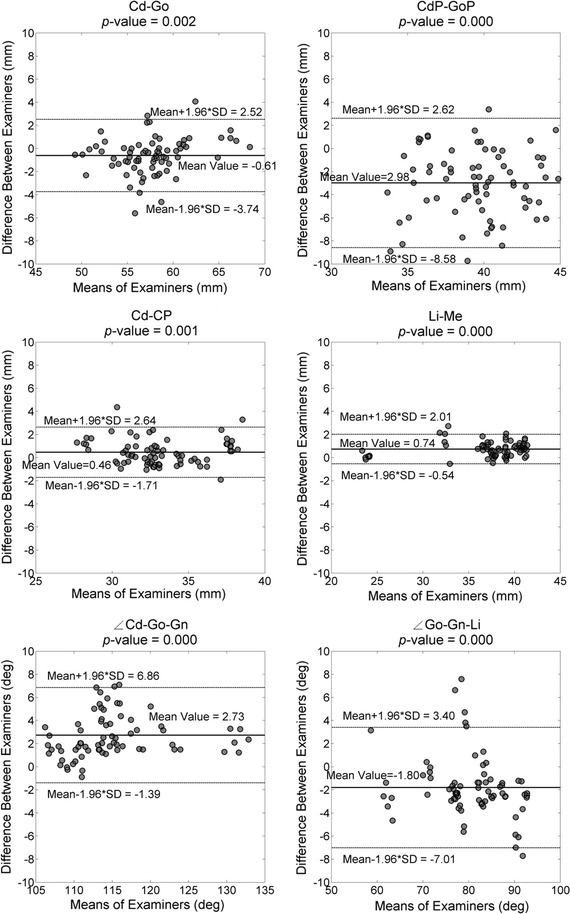
Bland & Altman plots of each of the mandibular parameter (Cd-GO, CdP-GOP, Cd-CP, Li-Me, Cd-GO-Gn, GO-Gn-Li) measurements by the two examiners for randomized head positions. The *solid lines* indicated the mean values of both examiners. The *dashed lines* indicated the 95% confident interval of the difference between the examiners

Very good inter-rater reliability was found for most parameters with ICC values greater than 0.89 but only good inter-rater reliability was found for CdP-GoP (ICC = 0.60) and Me-GoA (ICC = 0.70) (Table 2). There were significant differences between the examiners except for Go-Pog (p = 0.571), Cd-Go (p = 0.119) and Cd-CP (p = 0.074) (Table 2). Bland & Altman plots show that there were systematical biases between the examiners for most parameters, except for Go-Pog (Figs. 3, 4).

Very good inter-session reliability was found for most parameters in both examiners with ICC values greater than 0.84 but only good reliability by the junior examiner was found for CdP-GoP (ICC = 0.62, Table 3). Mean CVs of the parameters were all smaller than 0.13 for both examiners (Table 3). Between sessions, there were no significant differences in most parameters for the senior examiner, except for Go-Pog (p = 0.009), Cd-Go (p = 0.001) and Go-Gn (p = 0.006). However, significant between-session differences in most parameters were found for the junior examiner (Table 3).

**Table 3:** The mean (standard deviation) of each mandibular morphometric parameter for randomized head positions, as well as the inter-session reliability in terms of intra-class correlation coefficients (ICC) and coefficient of variation (CV) for the senior and junior examiner

	Senior					Junior				
	Mean_1_	Mean_2_	ICC	CV	*p* value	Mean_1_	Mean_2_	ICC	CV	*p* value
	(SD)_1_	(SD)_2_				(SD)_1_	(SD)_2_			
Cd-Gn	106.97 (4.88)	106.84 (5.02)	0.99	0.05	0.183	104.43 (5.28)	105.10 (5.28)	0.99	0.05	0.000*
Cd-B	95.05 (3.64)	94.98 (3.72)	0.98	0.04	0.327	93.64 (3.71)	93.63 (3.73)	0.98	0.04	0.450
Cd-Li	89.46 (4.16)	89.41 (4.33)	0.99	0.05	0.251	87.65 (3.82)	88.29 (3.85)	0.98	0.04	0.000*
Go-Pog	69.59 (4.42)	69.92 (4.26)	0.97	0.06	0.009*	69.47 (4.77)	71.19 (3.96)	0.92	0.07	0.000*
Cd-Go	57.36 (4.15)	56.87 (3.96)	0.96	0.07	0.001*	57.96 (3.87)	57.57 (4.30)	0.90	0.07	0.300
Go-Gn	68.55 (4.34)	68.93 (4.18)	0.97	0.06	0.006*	66.88 (4.92)	69.09 (4.12)	0.92	0.07	0.000*
CdP-GoP	38.02 (3.33)	37.70 (3.60)	0.84	0.09	0.267	41.00 (3.44)	39.62 (3.82)	0.62	0.08	0.001*
Me-GoA	58.14 (4.53)	58.01 (4.31)	0.89	0.08	0.600	54.38 (5.55)	56.41 (4.81)	0.84	0.10	0.000*
Cd-CP	33.38 (2.80)	33.47 (2.84)	0.98	0.08	0.222	32.91 (2.92)	33.14 (3.06)	0.97	0.09	0.187
Li-Me	37.53 (4.74)	37.61 (4.78)	1.00	0.13	0.328	36.79 (4.68)	36.81 (4.91)	0.99	0.13	0.655
∠Cd-Go-Gn	116.41 (6.54)	116.29 (6.67)	0.99	0.06	0.352	113.68 (6.38)	112.13 (6.07)	0.96	0.06	0.000*
∠Go-Gn-Li	79.51 (8.02)	79.29 (8.59)	0.99	0.10	0.124	81.31 (8.72)	81.21 (9.15)	0.98	0.11	0.219

The *p* values calculated from the paired *t* test are indicated with an asterisk when the differences in the measurement between examiners is less than 0.05

* Significant difference in the measurement between sessions

## Discussion

The current study aimed to determine the intra-rater, inter-rater and inter-session reliability of morphological measurements of the mandible with random head positioning errors on CBCT-synthesized cephalometric radiographs. With the CBCT-based DRR approach, the effects of the head positioning errors during imaging on the reliability of determining morphometric parameter on the synthesized cephalograms were studied without the effects of other factors such as superimposition of craniofacial structures.

The CBCT-based DRR approach is useful in many different radiographic studies [9, 23–25], the main advantage being that various study conditions can be simulated by using computer modeling techniques. This decreases the radiation exposure to the patient, which is the main disadvantage of radiographic methods. For the study of the effects of head positioning errors on the test–retest reliability of mandibular morphology measurements on cephalograms, a large number of CBCT-synthesized cephalograms were generated. If true cephalograms had been used, the patient would have received a large amount of radiation exposure, which is unacceptable. Moreover, with true cephalograms a definition of the precise 3D position of the head presents a great challenge. Although planar cephalograms or X-ray radiography appeared to be simpler at first glance, it is actually more difficult—if not impossible—to carry out accurately. Therefore, instead of a large number of true cephalograms, we used data from only a single 3D CBCT scan to generate the necessary synthesized cephalograms in 3D positions with random errors using the DRR technique. For the current purposes, the current DRR-based approach actually exposed the subject to much less radiation than using a large number of true cephalograms. The DRR approach can also simulate rare or clinically impossible conditions such as large degrees of head rotation, or can eliminate unfavorable anatomical structures. The current approach will also be useful for further studies examining the direction/axis of head positioning that may affect the results of malpositioning of the head during imaging, and the real life reliability for cephalogram-based measurements.

The effects of head positioning errors during imaging on the mandibular morphological measurement reliability were revealed by comparing the current results with those for a neutral position reported in the literature [9]. Very good intra-rater reliability and the same CV value (<0.13) were found for both examiners in measuring most of the mandibular parameters in the current study (Table 2). Similar results were also found for measurements in neutral head positions [9]. This suggests that head positioning errors during imaging had only very limited effects on the measurement reliability of a single examiner, regardless of their experience.

The measurements between the two examiners showed moderate to very good inter-rater reliability (ICC = 0.6–0.99, Table 3) in the current study, while good to very good inter-rater reliability (ICC = 0.62–0.99) was found for the neutral position of the mandible [9]. Bland & Altman plots also indicated systematic biases between the examiners on most of the parameters in the current study, whereas few parameters showed systematical biases for a neutral head position [9]. These results suggest that head positioning errors lead to slightly reduced reliability between examiners for most morphological parameters.

The junior examiner showed reduced inter-session reliability with many significant between-session differences for random head positioning errors when compared to the senior examiner (Table 3). For the neutral position without positioning errors, better inter-session reliability, i.e., good to very good reliability (ICC = 0.74–1.00), was reported for both junior and senior examiners [9]. These results showed that head positioning errors reduced the reliability of the morphological measurements based on landmark identification on the cephalometric radiography performed during different sessions, which could be compensated for by more clinical experience.

The current results provide new data to show that clinical assessment of the same patient within a single session by the same physician is generally reliable but longitudinal comparisons of the measurements on the same patient or comparisons between physicians should be made with care. Previous studies have shown significant differences in cephalometric tracing between examiners with different levels of clinical experience [34–36]. These results have significant relevance to the study of mandible growth, especially because the growth of the mandible is anisotropic and non-homogeneous within the bone, and non-linear over time [30]. However, these studies did not report how clinical experience of examiners would affect the measurement reliability. The current results suggest that variations in the measurement accuracy between examiners and/or over different time periods can lead to errors in the monitoring of the mandible growth. It is therefore suggested that cephalographic measurements for the study of mandibular growth should be made by the same experienced examiner.

The current results were limited to 3° of random rotational head positioning errors. Within this range good to very good intra-rater and inter-rater reliability could be achieved, and similar results for the inter-session reliability could be achieved by more experienced examiners. It is expected that with rotation angles greater than 3°, the measurement reliability would be further reduced. Nonetheless, the choice of 3° was considered to be reasonable because positioning the head with the assistance of the cephalostat often has errors less than 5° [13]. Further investigations using the current DRR-based approach will be needed to evaluate the individual effects of the three axes on the morphological measurements. In addition, the current results were obtained from CBCT-synthesized cephalograms based on a single commercially available cephalogram system. Further study will be needed to confirm the current findings on other cephalogram systems.

## Conclusions

For random head positioning errors good to very good intra-rater, inter-rater and inter-session reliability in measuring mandibular morphological parameters could be achieved by both examiners. However, higher inter-session reliability can be achieved by more experienced examiners. The results suggest that cephalographic measurements for the study of mandibular growth should be made by the same more experienced examiner because the growth of the mandible is anisotropic and non-homogeneous within the bone, and non-linear over time. The current DRR-based approach will be useful for evaluating individual factors that affect the morphological measurements.

## Abbreviations

CBCT: cone-beam computed tomography
DRR: digitally reconstructed radiography
ICC: intra-class correlation coefficient
3D: three-dimensional
2D: two-dimensional
ACS: anatomical coordinate system
CV: coefficients of variation
Cd: the most protruding point on the top of the mandibular condyle
CdP: the most posterior protruding point of the mandibular condyle
GOP: the most posterior protruding point of the ramus above the gonion
GO: the midpoint of the contour connecting the ramus and the body of the mandible
GOA: the most protruding point of the mandible before the gonion
Me: the center of the inferior border on the mandibular symphysis
Pog: the most anterior point on the contour of the chin
Gn: the center of the inferior border on the mandibular symphysis
B: the innermost point on the contour of the mandible between the incisor and the bony chin
Li: the incisal edge of the mandibular central incisor
CP: the top point of the coronid process
